# Differential regulation of chloride homeostasis and GABAergic transmission in the thalamus

**DOI:** 10.1038/s41598-018-31762-2

**Published:** 2018-09-17

**Authors:** Tobias Schmidt, Nikoo Ghaffarian, Camille Philippot, Gerald Seifert, Christian Steinhäuser, Hans-Christian Pape, Peter Blaesse

**Affiliations:** 1 Institute of Physiology I, University Hospital Münster, Münster, Germany; 2 Department of Anesthesiology, Intensive Care and Pain Medicine, University Hospital Münster, Münster, Germany; 3 Institute of Cellular Neurosciences, Medical Faculty, University of Bonn, Bonn, Germany

## Abstract

The thalamus is important for sensory integration with the ventrobasal thalamus (VB) as relay controlled by GABAergic projections from the *nucleus reticularis thalami* (NRT). Depending on the [Cl^−^]_i_ primarily set by cation-chloride-cotransporters, GABA is inhibitory or excitatory. There is evidence that VB and NRT differ in terms of GABA action, with classical hyperpolarization in VB due to the expression of the Cl^−^ extruder KCC2 and depolarizing/excitatory GABA action in the NRT, where KCC2 expression is low and Cl^−^ accumulation by the Cl^−^ inward transporter NKCC1 has been postulated. However, data on NKCC1 expression and functional analysis of both transporters are missing. We show that KCC2-mediated Cl^−^ extrusion set the [Cl^−^]_i_ in VB, while NKCC1 did not contribute substantially to Cl^−^ accumulation and depolarizing GABA action in the NRT. The finding that NKCC1 did not play a major role in NRT neurons is of high relevance for ongoing studies on the therapeutic use of NKCC1 inhibitors trying to compensate for a disease-induced up-regulation of NKCC1 that has been described for various brain regions and disease states like epilepsy and chronic pain. These data suggest that NKCC1 inhibitors might have no major effect on healthy NRT neurons due to limited NKCC1 function.

## Introduction

The thalamus represents a pivotal station in processing of sensory information in the central nervous system (CNS). The nuclei of the ventrobasal thalamus (VB) receive peripheral input and project to the somatosensory cortex. This relay function of the VB is modulated and controlled by projections from the *nucleus reticularis thalami* (NRT), which are exclusively mediated by gamma-aminobutyric acid (GABA), the main inhibitory transmitter in the CNS^1^. Thalamocortical dysrhythmia (TCD) has been proposed as a common mechanism in chronic pain, tinnitus, schizophrenia and epilepsy^2,3^. Moreover, a disturbed thalamic synchrony and GABAergic transmission have been linked to pathological conditions like absence epilepsy^4,5^. The binding of GABA to GABA receptors of the type A (GABA_A_R) opens Cl^−^ channels, thereby allowing Cl^−^ ions (and to a minor degree HCO_3_^−^ ions) to flow across the cell membrane. The direction and the amplitude of the Cl^−^ current depend on the intracellular chloride concentration [Cl^−^]_i_, which is determined by the action of cation-chloride cotransporters (CCC)^6–10^. Several CCC isoforms are known, but in mature neurons, mostly the K^+^-Cl^−^-cotransporter 2 (KCC2) maintains a low [Cl^−^]_i_ by mediating an outward directed Cl^−^ transport^11^. Under these conditions, the opening of Cl^−^ channels leads to an influx of Cl^−^ and consequently to a hyperpolarization. In immature neurons KCC2 is either not present or functionally inactive and a Na^+^-K^+^-2Cl^−^-cotransporter 1 (NKCC1)-mediated inward directed Cl^−^ transport dominates (e.g.^12,13^). In this case, the relatively high [Cl^−^]_i_ results in an efflux of Cl^−^ when GABA_A_Rs are activated, which depolarizes and sometimes even excites the postsynaptic neuron. Differences in GABA reversal potentials (E_GABA_) between VB and NRT (more negative E_GABA_ values in VB) have been demonstrated, though without knowledge of specific transporter action^14^. In some TCD, alterations in the expression and function of CCC have been described. For instance, chronic pain is associated with a downregulation of KCC2 protein expression in the thalamus of rats^15^, and genetically encoded impairment of KCC2 has been related to the pathogenesis of epilepsy in humans^16–18^. Furthermore, experimental manipulation of NKCC1 and KCC2 has been shown to either inhibit or promote seizure activity or alleviate chronic pain (e.g.^19–26^). In view of this, pharmacological targeting of CCC has become an attractive therapeutic option for the treatment of neonatal seizures, which are often resistant to classical anticonvulsants, and a variety of other neurological and psychiatric disorders (for review see^27–31^).

In the mature CNS there are only few – cellular and regional – examples, where KCC2 is absent or functionally inactive under physiological condition, indicating that GABA might be excitatory in these neurons. Cortical and hippocampal neurons exhibit a differential distribution of NKCC1 and KCC2 in different subcellular compartments, resulting in dominating NKCC1-mediated Cl^−^ transport and depolarizing GABA action in the axon initial segment (AIS)^32–34^. On a regional level, KCC2 expression is low in the NRT^35,36^, but it is unknown, if NKCC1 contributes to Cl^−^ homeostasis in NRT neurons. In addition there are also controversial results whether or not GABA action in the NRT is inhibitory^14,37,38^ or excitatory^36,39^.

To decipher the molecular mechanisms of Cl^−^ homeostasis in the thalamus is crucial for a better understanding of TCD and the development of new therapeutic strategies. However, the role of NKCC1 and KCC2 in Cl^−^ homeostasis and consequently in GABAergic transmission in the thalamus is poorly understood. In this study, we hypothesized that KCC2 is responsible for Cl^−^ extrusion in VB, while NKCC1 accumulates Cl^−^ in NRT neurons. We show that the high expression levels of KCC2 in VB relate to E_GABA_ values negative from resting membrane potential. Using single-cell RT-PCR, we demonstrate the presence of NKCC1 mRNA in the NRT, although perforated patch approaches indicate that NKCC1 is not responsible for Cl^−^ uptake. In addition, our results provide further evidence for depolarizing GABA action in the NRT.

## Results

### Differential protein expression of KCC2 and NKCC1 in VB and NRT

**Figure 1:**
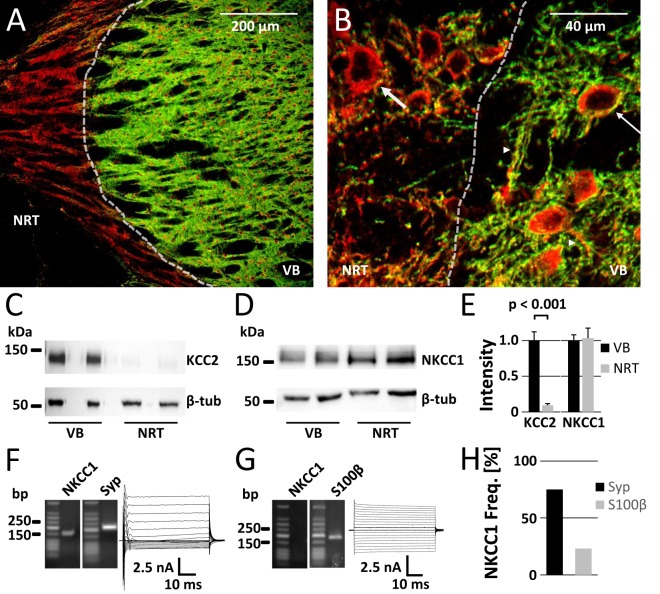
Differential expression of KCC2 and NKCC1 in VB and NRT detected by immunostaining and Western blot analysis. (**A**) KCC2 (green) protein expression was high in VB, whereas it was barely detectable in the NRT. MAP2 was used as a somato-dendritic marker (red). The same distribution was also visible at the cellular level in the transition zone between VB and NRT (**B**), where MAP2-positive somata (thin arrow) and dendrites (arrow heads) in VB were labelled by KCC2 immunoreactivity (KCC2-IR), while KCC2-IR was not detectable around somata in the NRT (thick arrow). KCC2-positive dendrites in the NRT originated presumably from thalamo-cortical VB neurons. (**C**) KCC2 was present in immunoblots with samples of VB tissue, but not in NRT samples. β-tubulin (β-tub) was used as loading control. (**D)** NKCC1 was expressed ubiquitously in VB and NRT. **(E)** Quantification of KCC2- and NKCC1-immunoreactivity. Protein expression of KCC2 in NRT reached 9% of the level seen in VB (n = 7), whereas NKCC1 levels did not differ between VB and NRT (n = 6; paired Student’s t-test). (**F**) Agarose gel images showing NKCC1 transcript expression and respective current pattern of a NRT neuron after de- and hyperpolarization of the cell. Synaptophysin (Syp) served as positive control for neuronal mRNA detection. Expected product lengths were 159 bp for NKCC1 and 215 bp for synaptophysin. (**G)** Agarose gel and respective current pattern of a NRT astrocyte. S100β (product length: 186 bp) served as positive control for astrocytal mRNA detection. (**H**) NKCC1 mRNA was present in most NRT neurons (30 out of 40 Syp-positive cells) but to a lesser extent in NRT astrocytes (3 out of 13 S100β-positive cells).

Previous studies showed that KCC2 is strongly expressed in the VB, whereas it is barely detectable by immunostaining in the NRT^35,36^. To confirm the differential protein expression, we first analyzed immunoreactivity of KCC2 (KCC2-IR) in VB and NRT (Fig. 1A). On a cellular level VB neurons exhibited a KCC2 signal at both somata and dendrites, while NRT neurons did not show KCC2-IR (Fig. 1B). Consistent with the immunostaining, Western blot analysis detected KCC2-IR in samples from the VB but only a very low level in NRT samples (9% when normalized to VB; Fig. 1C,E). In contrast, an NKCC1 signal was present in VB and NRT samples with similar intensity (Fig. 1D,E). While KCC2 is a neuron-specific transporter, NKCC1 can be found in neurons and glial cells^40–42^. To support a potential role of NKCC1 in neuronal Cl^−^ accumulation in the NRT, we tested for the neuronal expression of NKCC1. Unfortunately, we failed to produce a reliable immunostaining for NKCC1 with commercially available antibodies (see also^6^). Therefore, single-cell RT-PCR was used to distinguish between neuronal and glial NKCC1 expression in the NRT. Cytosol from single cells located in the NRT from a hGFAP/eGFP-coexpressing mouse line that enabled discrimination of neurons and astrocytes was selectively collected and used for RT-PCR^43,44^. NKCC1 mRNA could be detected in 30 of 40 (75%) NRT neurons and in 3 of 13 (23.1%) NRT astrocytes. This suggested that NKCC1 is expressed in a majority of NRT neurons (Fig. 1F–H).

### NRT neurons exhibit a more depolarized GABA reversal potential than VB neurons

**Figure 2:**
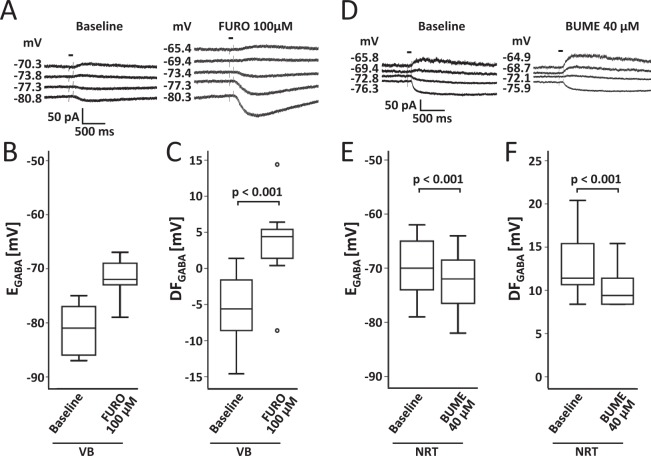
NRT neurons exhibited more positive GABA reversal potentials than VB neurons. (**A**,**B)** Perforated patch recordings revealed KCC2-mediated transport in VB neurons. Bath application of the KCC2 inhibitor furosemide (FURO; in the presence of 10 µM bumetanide) shifted E_GABA_ to more positive values (n = 13; paired Student’s t-test). (**C)** Accordingly, FURO shifted the GABA driving force (DF_GABA_) to more positive values. (**D,E)** NRT neurons exhibited native values of E_GABA_ which were more positive than those of VB neurons and similar to VB neurons after FURO application. Bath application of the NKCC1 inhibitor bumetanide (BUME) induced a slight (although significant) shift to more negative E_GABA_ values and reduced DF_GABA_ **(F**) (n = 15; paired Student’s t-test). Sample traces (**A,D**) show representative current traces in response to command potential steps of single neurons before and after bath application of FURO or BUME, respectively (bar indicates GABA puff application).

It is not known so far, if the differential expression of KCC2 has any functional impact on Cl^−^ homeostasis and E_GABA_ in the VB-NRT system. Using the gramicidin perforated patch-clamp technique, which allows measurements without compromising native [Cl^−^]_i_, we examined E_GABA_ under control conditions (Fig. 2). The resting membrane potential was −75.5 ± 1.1 mV in VB neurons and −82.7 ± 1.4 mV in NRT neurons. E_GABA_ values in VB neurons were more negative than in NRT neurons (−81.4 ± 1.2 mV vs. −69.7 ± 1.4 mV; p < 0.001; Student’s t-test). To test if the more negative E_GABA_ value of VB neurons is due to KCC2-mediated Cl^−^ extrusion we applied the loop diuretics bumetanide and furosemide to pharmacologically isolate NKCC1 and KCC2, respectively. Bumetanide specifically inhibits NKCC1 at low concentrations (10 µM), whereas furosemide (and bumetanide in higher concentrations >10 µM) inhibits both transporters. Bath application of furosemide (in the presence of 10 µM bumetanide) shifted E_GABA_ of VB neurons by 9.6 mV to more positive values (Fig. 2A; −71.8 ± 1.0 mV; p < 0.001; paired Student’s t-test). The more positive E_GABA_ values of NRT neurons suggest the presence of a Cl^−^ uptake mechanism. In initial experiments with a standard concentration of bumetanide of 10 µM, E_GABA_ was not affected. To exclude submaximal inhibition of NKCC1, we performed an additional series of experiments using 40 µM bumetanide. At this higher concentration, bumetanide induced a small (but statistically significant) shift of 2.6 mV to more negative E_GABA_ values (Fig. 2B; −72.4 ± 1.4 mV; p < 0.001; paired Student’s t-test).

### KCC2-mediated Cl^−^ extrusion is present in VB, but not in NRT neurons

**Figure 3:**
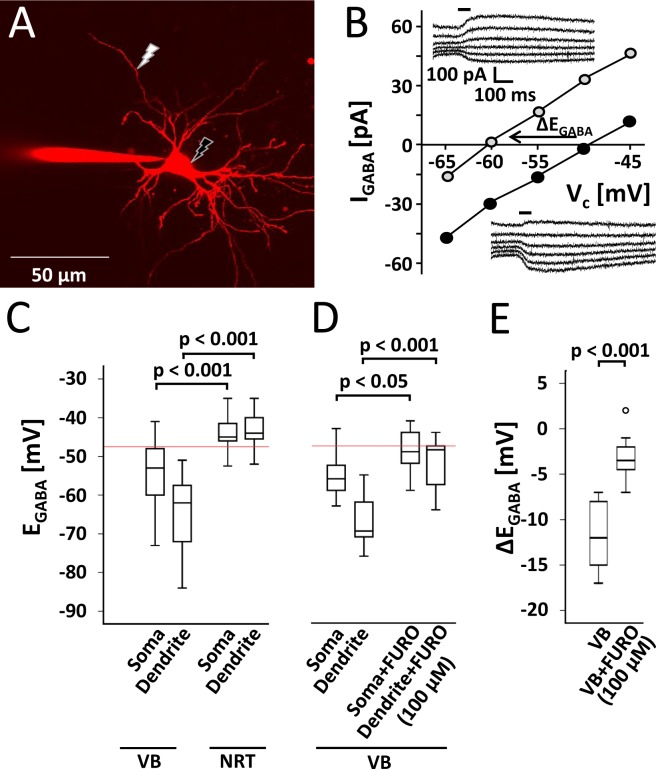
KCC2-mediated Cl^−^ extrusion is present in VB, but not in NRT neurons. **(A)** Superimposed image stack of a VB neuron loaded with a defined Cl^−^ concentration and Alexa 594 as a fluorescent dye. GABA was locally uncaged at the indicated positions (flash). (**B)** Exemplary I-V-curves recorded from a VB neuron with subsequent GABA uncaging at the soma (filled circles; lower inlay) and at the dendrite in 50 µm distance from the soma (open circles; upper inlay). Currents were recorded at command potentials (V_C_) steps of +5 mV starting from −70 mV (inset: bar represents GABA photolysis). KCC2 shifted the imposed GABA reversal potential (E_GABA_; defined by the X-intercept) at the dendrite to more negative values compared to the soma by extruding Cl^−^, since the slow diffusion of Cl^−^ from the pipette via the soma is not able to fully counteract KCC2-mediated Cl^−^ extrusion in dendrites. (**C)** Whole cell recordings revealed that imposed E_GABA_ values of VB neurons at both soma and dendrite were more negative compared to NRT neurons (n = 17 and n = 15 at the soma, n = 13 and n = 7 at the dendrite, respectively; Student’s t-test). Moreover VB neurons exhibited gradients between soma and dendrite. In addition E_GABA_ values of VB neurons are hyperpolarized from the calculated reversal potential of Cl^−^ (−47.5 mV; illustrated by the red line), which is determined by the pipette solution (20 mM Cl^−^). The hyperpolarization compared to the NRT and to the calculated Cl^−^ reversal potential and somato-dendritic gradients of the VB together indicate a strong KCC2 activity. In the NRT, where KCC2 was not detectable, E_GABA_ values at the soma as well as at the dendrite were close to calculated reversal potential and no somato-dendritic gradients were found. (**D**) In a new set of experiments bath application of the KCC2 inhibitor furosemide (FURO; in the presence of 10 µM bumetanide) shifted the imposed E_GABA_ of VB neurons towards the calculated reversal potential at the soma and even more at the dendrite (n = 10 and n = 8 for control and FURO, respectively; Student’s t-test). Furthermore, FURO induced a significant decrease in somato-dendritic gradients (ΔE_GABA_) in VB neurons (n = 10 and n = 8, respectively; Student’s t-test) (**E**).

To study KCC2-mediated transport capability under a constant Cl^−^ load, we used a defined Cl^−^ concentration (20 mM) in whole cell recordings and examined the imposed E_GABA_ values at somata and dendrites^45^. By extruding Cl^−^, KCC2 is capable of shifting the imposed E_GABA_ to more negative values compared to the calculated E_GABA_ defined by the Cl^−^ concentration of the pipette solution. While somatic E_GABA_ values are mainly defined by the Cl^−^ concentration of the pipette solution due to fast dialysis between the pipette solution and the intracellular milieu, E_GABA_ values at the dendrite are more negative than those at the soma, because the diffusion of Cl^−^ from the pipette via the soma is not able to fully counterbalance KCC2-mediated Cl^−^ extrusion in dendrites. Thus, somato-dendritic gradients serve as a quantitative measure for KCC2 activity^45^ (Fig. 3A,B). In agreement with perforated patch experiments, E_GABA_ values of VB neurons were hyperpolarized from the calculated E_GABA_ (indicated by the red line) and exhibited more negative E_GABA_ values at the soma compared to neurons of the NRT (Figs. 3C; −54.4 ± 2.3 mV vs. −44.0 ± 1.3 mV; two-way ANOVA: F_1,48_ = 45.329, p < 0.001 for VB vs. NRT, F_1,48_ = 3.844, for soma vs. dendrite, p = 0.056; F_1,48_ = 5.460, p < 0.05 for main effect interaction VB/NRT*soma/dendrite; post hoc Student’s t-test, p < 0.001, respectively). These data indicated a robust KCC2 activity strong enough to overcome the constant Cl^−^ load via the patch pipette. Accordingly, E_GABA_ values of the dendrite were even more negative in VB neurons compared to the NRT (−64.6 ± 2.5 mV vs. −43.1 ± 1.9 mV; post hoc Student’s t-test, p < 0.001), resulting in somato-dendritic gradients (ΔE_GABA_) of −9.2 ± 1.1 mV. In NRT neurons, which lack KCC2, E_GABA_ values of both somata and dendrites were close to the calculated E_GABA_ and did not exhibit somato-dendritic gradients (−0.57 ± 3.96 mV), demonstrating the reliability of the experimental approach. In VB neurons, bath application of the KCC2 inhibitor furosemide (100 µM) shifted E_GABA_ to more positive values (Fig. 3D; −55.0 ± 1.8 mV vs. −48.8 ± 1.9 mV at the soma; −66.1 ± 1.8 mV vs. −51.9 ± 2.3 mV at the dendrite; two-way ANOVA: F_1,32_ = 26.187, p < 0.001 for control vs. FURO; F_1,32_ = 12.001, p < 0.01 for soma vs. dendrite; F_1,32_ = 3.995, p = 0.054 for main effect interaction control/FURO*soma/dendrite; post hoc Student’s t-test, p < 0.05 and p < 0.001, respectively), and significantly diminished the somato-dendritic gradients (Fig. 3E; −11.1 ± 1.0 mV vs. −3.1 ± 0.9 mV; p < 0.001; Student’s t-test). In summary, these results show that KCC2 is responsible for Cl^−^ extrusion in the VB, whereas KCC2 activity is not detectable in the NRT.

### Inhibiting NKCC1 did not restrain neuronal Cl^−^ uptake in the NRT

**Figure 4:**
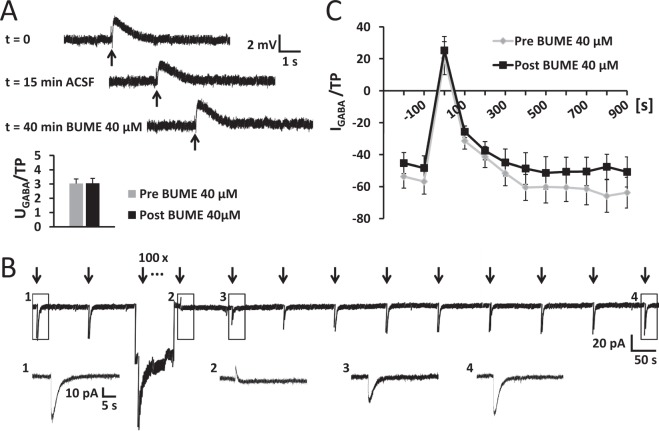
Inhibition of NKCC1 did not restrain neuronal Cl^−^ uptake in the NRT. **(A)** Example of a NRT neuron (V_M_ = -91.4 mV) responding to local GABA puff application (indicated by bar). Under perforated patch conditions in current clamp, all recorded NRT neurons were depolarized from resting membrane potential (I = 0 pA). Application of the NKCC1 inhibitor bumetanide (BUME) did not alter the amplitude of the GABA response (U_GABA_/TP: voltage peak amplitude normalized to test pulse; p = 0.924 for n = 14; paired Student’s t-test). (**B**) In voltage clamp, depletion of intracellular Cl^−^ was achieved by application of 100 GABA puffs during hyperpolarization of the cell to −100 mV. GABA puffs were depolarizing, resulting in negative current responses. The first response to GABA immediately after the depletion was hyperpolarizing, thus the recorded current turned positive. Thereafter, GABA responses recovered to baseline level (magnification of boxed parts depicted in insets). GABA puffs are marked by arrows. (**C)** After a complete depletion protocol (Pre BUME) as shown in (**B**), bumetanide was bath applied for 20 min, before a second depletion protocol was performed in the same neuron (Post BUME). BUME application did neither significantly alter the time course nor the amplitude of the GABA responses, which suggests that NKCC1 is not responsible for Cl^−^ uptake in NRT neurons (repeated measures ANOVA: p = 0.407 and p = 0.331, respectively, for n = 6, see text for detailed description).

The application of the NKCC1 inhibitor bumetanide (40 µM) under steady-state conditions in perforated patch recordings revealed only a small E_GABA_ shift of 2.6 mV (Fig. 2). At resting membrane potential (zero net membrane current), GABA action was depolarizing in every NRT neuron recorded (n = 14; Fig. 4A). The application of bumetanide (40 µM) did neither switch the depolarizing responses to hyperpolarizing nor decrease the amplitude of the depolarizing response (Fig. 4A; p = 0.924; paired Student’s t-test). To test the functional relevance and Cl^−^ accumulation capacity of NKCC1 in NRT neurons, we performed recordings using a Cl^−^ depletion protocol that challenges the transporter by decreasing [Cl^−^]_i_^46^. Depletion of [Cl^−^]_i_ was achieved by applying a series of GABA puffs while cells were kept at −100 mV (Fig. 4B). The first GABA application following the depletion was hyperpolarizing, indicated by a switch from negative to positive current responses, which suggested a successful depletion and a significant fall in [Cl^−^]_i_. After this initial switch, GABA responses returned to baseline values indicating recovery of the [Cl^−^]_i_. To test whether or not NKCC1 is responsible for Cl^−^ accumulation, we applied the NKCC1 inhibitor bumetanide (40 µM) via the bath for 20 min, followed by a second depletion protocol in the same neuron. Neither the time course of the recovery nor the amplitude of the GABA responses showed any significant difference in presence of bumetanide compared to the depletion under control conditions (Fig. 4C; repeated measures ANOVA: F_1,10_ = 0.750, p = 0.407 for main effect BUME and repeated measures ANOVA: F_1,5_ = 1.156, p = 0.331 for main effect amplitude difference, normalized to test pulse, respectively). Taken together, these data indicate that NKCC1 function seems not to play a major role in Cl^−^ uptake in NRT neurons.

## Discussion

Previous studies revealed a differential protein expression of KCC2 in VB and NRT and NKCC1 was hypothesized to be responsible for Cl^−^ accumulation and depolarizing/excitatory GABA action in the NRT^35,36^. However, a detailed characterization of the respective transporter function in the VB-NRT system was missing. We demonstrate that the differential protein expression of KCC2 is functionally relevant, meaning that KCC2 transport activity is present in VB but not in NRT neurons with consequently more negative E_GABA_ values in VB neurons. The very low KCC2 protein level (9% in NRT when compared to VB) and the absence of effects of KCC2 inhibition in functional assays in the NRT is in contrast to a recent study^38^. By quantification of KCC2 immunostainings, Klein *et al*. (2018) demonstrated a KCC2 protein level in the NRT of >50% of the VB protein level in rats and mice (20 days of age and older). In addition, the authors showed regional differences within the NRT, with low KCC2 protein levels in the center and slightly higher protein levels in the anterior and posterior parts of the NRT. For rat NRT neurons, there is evidence that KCC2 is functionally relevant^38^. If the absence of KCC2 function in our functional assays is explained by regional differences has to be addressed in further studies. In the present sets of experiments, we did not find evidence for such regional differences. This might be partly due to the fact that in the transverse slice preparation used, slices with a clearly identifiable NRT from the center of the NRT have been preferred to avoid contamination with non-NRT neurons.

While NKCC1 transcripts could be detected in 75% of NRT neurons, we were not able to provide evidence for a major contribution of NKCC1 to neuronal Cl^−^ uptake in the NRT. At a relatively high concentration of 40 µM, the NKCC1 inhibitor bumetanide induced a small shift of 2.6 mV in E_GABA_, but had neither an effect on the amplitude of GABA evoked depolarizing responses of NRT neurons nor did it affect the kinetics of the recovery of the [Cl^−^]_i_ after a Cl^−^ depletion. This is in contrast to studies demonstrating a Cl^−^ accumulating effect of NKCC1 in CA3 pyramidal neurons^47^, Cajal-Retzius cells^46^, cochlear nucleus neurons^48^ and Gonadotropin-releasing hormone expressing neurons^49^, but in line with findings in retinal neurons^50^. However, the experiments demonstrating NKCC1-mediated Cl^−^ accumulation were performed in cells from immature embryonic or newborn mice and rats, in which the NKCC1/KCC2 developmental shift is not completed and NKCC1 seems to be the dominating transporter^7^. To our knowledge the only example of bumetanide efficacy in more mature (adolescent) rodents on a (sub-)cellular level is the AIS of cortical and hippocampal neurons^33^. But even in the AIS NKCC1-mediated Cl^−^ transport and, thereby, excitatory or depolarizing GABA responses seem to disappear rapidly after postnatal day 20^34^. In our study, using adolescent/adult (21–60 days old) mice and a relatively high concentration of bumetanide, we could not detect relevant Cl^−^ accumulation mediated by NKCC1. Whether there are other (not yet identified) Cl^−^ accumulators or anion exchangers (AE) responsible for the high [Cl^−^]_i_ needs to be further studied^6,49^. Rahmati *et al*. (2017) discussed a list of putative candidates for Cl^−^ uptake (e.g., AE2, AE3 and other members of the solute carrier family like Slc26A7 and SlcA11)^51^. Publicly available expression data in the Allen Mouse Brain Atlas indicates expression of several of these candidates in the NRT^52^. Therefore, an in depth analysis of the expression and function is needed to identify the responsible Cl^−^ uptake mechanism(s).

Previously, GABA action and synaptic transmission within in the NRT was thought to be inhibitory, while detected excitatory responses were attributed to electrical coupling^14,37,53^. Excitatory GABA action in the NRT was primarily observed *in vivo*^39^, but later on described *in vitro* as well^36^. While a recently published computational model indicates that rat NRT neurons switch from GABA-mediated inhibition to GABAergic excitation in an activity-dependent manner^38^, our study provides further evidence for depolarizing/excitatory GABA action in the NRT under steady-state conditions. In addition, we prove that this depolarizing/excitatory GABA action is not NKCC1-dependent.

Based on the numerous findings that various neurological disease states are accompanied (and perhaps even triggered) by a downregulation of KCC2 and an upregulation of NKCC1, compounds modulating these transporters have been extensively studied in animal models and in several clinical trials (for review see^30,31,54^). The NKCC1 inhibitor bumetanide attracted most attention with some promising results, but also controversial data on efficacy, mode of action and pharmacokinetics^27,30^. The data on KCC2 and NKCC1 expression and function in the thalamus presented here provide a better understanding of the action of CCC modulators. Particularly the finding that NKCC1 seems not to play a major role under physiological conditions in adolescent/adult NRT neurons indicates that the effect of bumetanide (or even more efficient bumetanide derivatives) might be restricted to neurons with a disease-related upregulation of NKCC1 (e.g., cortical neurons after traumatic brain injury^55^) without affecting healthy NRT neurons.

## Methods

All animal experiments have been approved by local authorities (LANUV NRW, Recklinghausen, Germany) and fulfill the requirements of the European Directive 2010/63/EU. Experiments were performed in 21–60 days old C57Bl/6 J male mice and human glial fibrillary acidic protein-enhanced green fluorescent protein mice^56^ (hGFAP-eGFP-mice) obtained from Envigo (Horst, Netherlands) or in-house breeding. Mice were held at a circadian rhythm with a light-dark cycle of 12:12 h, with lights on at 6:00 AM and access to food and water *ad libitum*. As there was no evidence for age-dependent differences, data from 21–60 days old mice were pooled for analysis.

### Slice preparation and immunoblots

Procedures were performed as previously described^57^. Mice were deeply anesthetized with isoflurane (3.5 vol%) and decapitated. Brains were rapidly removed and put into oxygenated (95% O_2_/5% CO_2_) and ice-cold solution for slicing, containing (in mM) 2.5 KCl, 1.25 NaH_2_PO_4_, 10 MgSO_4_, 0.5 CaCl_2_, 20 PIPES, 10 glucose and 200 sucrose; pH was 7.35, osmolarity ∼300 mOsm/kg. Coronal slices (250–300 μm thick) were prepared on a vibratome (Leica Microsystems, Wetzlar, Germany) in ice-cold solution. Slices were kept in an incubation solution containing (in mM) 125 NaCl, 2.5 KCl, 1.25 NaH_2_PO_4_, 24 NaHCO_3_, 2 MgSO_4_, 2 CaCl_2_ and 10 glucose (pH 7.4, osmolarity ∼300 mOsm/kg) at 34 °C for 20 min and then allowed to equilibrate for at least 1 h at room temperature. For recordings, slices were transferred to a recording chamber and continuously superfused with artificial cerebrospinal fluid (ACSF; ∼2 ml/min) containing (in mM) 120 NaCl, 2.5 KCl, 1.25 NaH_2_PO_4_, 22 NaHCO_3_, 2 MgSO_4_, 2 CaCl_2_ and 25 glucose; pH 7.4, osmolarity ∼290 mOsm/kg.

For immunoblots, VB and NRT tissue was manually excised from slices under visual control using a binocular microscope and immediately frozen to −80 °C. Tissue samples were homogenized in lysis buffer (150 mM NaCl, 1% Triton, 50 mM Tris-BASE) with a protease inhibitor cocktail (Complete Ultra Tablets, Mini, Roche, Basel, Switzerland). Total protein was determined with Pierce BCA Protein Assay Kit (Thermo Fisher Scientific, Waltham, MA, USA). Protein samples in loading buffer (5x Protein Loading Buffer, Fermentas, Waltham, MA, USA) were separated by SDS-PAGE in a 5%/7.5% SDS-2-phase gel and sample buffer (25 mM Tris-BASE, 0.1% SDS, 192 mM glycine). After separation, proteins were transferred to a nitrocellulose membrane using the Trans-Blot Turbo Transfer System (BioRAD, Hercules, CA, USA) and blocked in TBST/5% milk (in mM; 24.8 Tris, 136.9 NaCl, 2.68 KCl, 0.1% Tween 20, and 5% nonfat dry milk, pH 7.5) for 2.5 h at room temperature. Membranes were divided at 75 kDa and incubation with the primary antibodies (rabbit-anti-KCC2, 1:5000, Merck-Millipore, Darmstadt, Germany (No. 07-432); mouse-anti-NKCC1 (T4), 1:2000, Developmental Studies Hybridoma Bank (DSHB), T4 was deposited to the DSHB by Lytle, C./Forbush III, B. (DSHB Hybridoma Product T4), Iowa City, IA, USA (No. AB 528406); rabbit-anti-β-tubulin, 1:5000, Covance, Princeton, NJ, USA (No. PRB-435P)) in TBST/1% milk was performed overnight at 4 °C with agitation. The secondary antibodies (goat-anti-rabbit HRP, 1:1000, Dako, Glostrup, Denmark (No. P0448) and goat anti-mouse HRP, 1:1000, Dako (No. P0447)) in TBST/1% milk were applied for 1 h at room temperature with agitation. Immunoreactivity was detected using a chemoluminescence kit (Super Signal West Femto Maximum Sensitivity Substrate, Thermo Scientific, No. 34095) and the BioRAD Chemidoc system (Figs S1 and S2). For quantification, samples were normalized to the corresponding β-tubulin loading control.

### Electrophysiological recordings and data analysis

Patch clamp recordings were made at 30 °C from the VB and NRT with an EPC-10 single patch-clamp amplifier (HEKA Elektronik, Lambrecht/Pfalz, Germany). Neurons were visualized by infrared video microscopy (Olympus BX51WI, Olympus, Tokyo, Japan). Pipettes had a tip resistance of 2–4 MΩ, series resistance (R_s_) was ≤12 MΩ. In whole cell recordings, cells were discarded from analysis when the resting membrane potential was more positive than −50 mV after breaking in. For determination of GABA reversal potentials patch pipettes were filled with an intracellular solution containing (in mM) 120 K-Gluconate, 20 KCL, 10 HEPES, 10 Phosphocreatin, 4 Mg-ATP, 0.3 Na-GTP and Alexa Fluor 594 hydrazide; pH 7.25 adjusted with 1M KOH. After establishing a steady-state and visualization of cell morphology by laser-scan microscopy (Olympus Fluoview F3100), Ruthenium-bipyridine-triphenylphosphine caged GABA (RuBi-GABA, Tocris) was locally released either at the soma or at the dendrite by scanning a small region of interest (ROI; ∼1 μm diameter) as described previously^45,58^. The reversal potential values were corrected offline for the calculated liquid junction potential (10 mV).

In gramicidin perforated patch experiments^59^ pipettes were tip-filled with (in mM): 125 K-Gluconate, 5 KCl, 10 HEPES, 5 EGTA, 1 MgCl_2_, 0.5 CaCl_2_, pH 7.25 adjusted with 1 M KOH, and back-filled with the same solution containing 40 μg/ml gramicidin (Sigma). Gramicidin was dissolved in dimethyl sulfoxide (DMSO) (5 mg/100 µl), before diluting to the final concentration. Gramicidin containing solutions were freshly prepared and dissolved by ultrasound sonication. Resting membrane potential ranged from −55 mV to −67 mV in VB neurons and from −59 mV to −73 mV in NRT neurons after establishing a steady-state. Access resistance (R_A_) was continuously monitored online using a 10 mV test pulse with 10 ms duration and decreased from initial gigaseal to 50–150 MΩ after 30–45 min. To compensate for gramicidin-induced changes in R_A_ over time, current and voltage amplitudes in the depletion and current-clamp experiments, respectively, were normalized to the test pulse. Cells were discarded from analysis when R_A_ showed a sudden drop, indicating whole-cell access. In addition, accuracy of the perforated patch configuration was checked in some cells by application of Alexa Fluor 594 to the pipette solution. For determination of reversal potentials, data were corrected offline for R_A_ and for the calculated liquid junction potential (−14.4 mV).

In perforated patch experiments, reversal potentials were determined either by puff application of muscimol (150 µM) or uncaging of RuBi-GABA at varying command potentials. An inter-sweep interval of 60 s ensured equilibration of the [Cl^−^]_i_ between GABA/muscimol applications. Reversal potentials in puff and uncaging experiments did not differ and data were pooled. Puff application of GABA (150 µM) was used in the depletion experiments as indicated, because of the shorter equilibration time compared to muscimol.

Recordings were filtered at 3 kHz (8-pole Bessel filter), sampled at 10 kHz with Patchmaster software (HEKA Elektronik), and analyzed off-line with the program Fitmaster (HEKA Elektronik) and Excel (Microsoft, Redmond, WA, USA). All electrophysiological recordings were made in the presence of DNQX (10 µM, Abcam, Cambridge, UK), CGP55845A (2.5 µM, Tocris, Bristol, UK) and TTX (500 nM, Biotrend, Cologne, Germany; except in current clamp experiments) to block NMDA receptors, GABA_B_ receptors and voltage-gated Na^+^ channels, respectively. The NKCC1 inhibitor bumetanide (10–40 µM, Tocris) and the KCC2 inhibitor furosemide (100 µM, Ascent scientific, Cambridge, UK) were bath applied as indicated in the respective experiment.

### Immunohistochemistry

Mice were perfused transcardially under deep isoflurane anesthesia (5 vol%) with phosphate-buffered saline (PBS; pH 7.4) followed by 4% paraformaldehyde (in 150 mM Na-phosphate buffer, pH 7.4). Brains were removed and stored in 4% paraformaldehyde overnight at 4 °C. After incubation in 30% sucrose-PBS for cryoprotection, coronal sections (30 μm) were cut with a freezing microtome (Frigomobil 1205; Jung, Heidelberg, Germany). Sections were collected in 15% sucrose-PBS, followed by three washes in PBS. Within one week after cutting sections were blocked for 1 h in 3% bovine serum albumin (BSA), 10% goat serum, and 0.3% Triton in PBS. A guinea pig anti-MAP2 antibody against microtubule-associated protein 2 was obtained from Synaptic Systems (Göttingen, Germany, No. 188004) and a rabbit anti-KCC2 antibody was obtained from Merck-Millipore (No. 07-432). Antibodies were diluted 1:1000 in blocking solution. Incubation with agitation was carried out at 4 °C overnight. After three wash steps in PBS, sections were transferred to carrier solution (0.3% Triton, 1% BSA, 1% goat serum) and treated with the secondary antibodies goat anti-rabbit conjugated to Alexa Fluor 488 and goat anti-guinea pig conjugated to Alexa Fluor 594 (both diluted 1:1000, Life Technologies, Carlsbad, CA, USA) for 2 h at room temperature. After four additional washes in PBS, sections were mounted with Vectashield HardSet mounting medium (Vector laboratories, Burlingame, CA, USA) and imaged with a laser scanning confocal microscope (Nikon eC1plus) equipped with a CFI75 LWD × 16/0.8 NA objective (Nikon). Sequential two-channel imaging with 488- and 543-nm excitation in combination with adequate emission filters (515/30 nm and 605/75 nm, respectively) prevented bleed-through. Images (1024 × 1024 pixels) were further processed with ImageJ (http://rsb.info.nih.gov/ij/).

### Cell harvesting, reverse transcription and single-cell RT-PCR

Single-cell RT-PCR was performed as described previously^43,44^. After electrophysiological recording (de- and hyperpolarization of the cell membrane between +20 and −160 mV; holding potential −70 mV for neurons and −80 mV for astrocytes, respectively; 10 mV increments), the cell content was harvested by applying negative pressure to the pipette, while keeping the high seal resistance between pipette tip and membrane. The flow of the cytoplasm into the pipette was controlled via a CCD camera (Leica DFC350FX on top of a laser scanning microscope SP5, Leica, Wetzlar, Germany). Only single cells without any adherent tissue debris were harvested. To increase the specificity of harvesting and subsequent reverse transcription (RT)-PCR, only part of the cytoplasm was aspirated into the recording pipette. The cell content and about 3 µl of the pipette solution were expelled into a reaction tube containing 3 µl DEPC-treated water. The tube was frozen in liquid nitrogen and stored at −20 °C until RT. First strand buffer (Thermo Fisher Scientific), dNTPs (4 × 250 µM; Applied Biosystems, Darmstadt, Germany), RNasin™ (20 U; Promega, Mannheim, Germany), random hexamer primers (50 µM; Roche), and reverse transcriptase (Maxima, 100 U, Thermo Fisher) were added to the frozen cell content and this RT reaction mix was incubated at 37 °C for 1 h (final volume 10 µl).

A multiplex two round single cell PCR was performed with primers for NKCC1 and cell type-specific genes as a positive control (Table 1). The first PCR was performed after adding PCR buffer, MgCl_2_ (2.5 mM), primers (200 nM each for NKCC1; 100 nM each for S100β or synaptophysin, respectively) and 3.5 U *Taq* polymerase (Applied Biosystems, Karlsruhe, Germany) (final volume 50 µl). Thirty-five cycles or 20 cycles were run (denaturation at 94 °C, 25 s; annealing at 51 °C, 2 min for the first 5 cycles, and 45 s for the remaining cycles; extension at 72 °C, 25 s; final elongation at 72 °C, 7 min). An aliquot (2 µl) of the PCR product was used as a template for the second PCR (35 cycles; annealing at 54 °C, first 5 cycles: 2 min; remaining cycles: 45 s) using nested primers (Table 1). The conditions were the same as described for the first round, but dNTPs (4 × 50 µM) and Platinum *Taq* polymerase (2.5 U; Applied Biosystems) were added. Products were identified with gel electrophoresis using a molecular weight marker (Low molecular weight marker, New England Biolabs, Frankfurt, Germany). As a positive control, RT-PCR for total RNA isolated from mouse brain was run in parallel. Negative controls were performed using distilled water (Figs S3 and S4).

**Table 1:** Primers used for single cell RT-PCR.

Gene	Sequence	Position	Product length	Accession No.
NKCC1	se 5′-TGGCCGTTGCTATGTATGTTGTCG as 5′-GAAAATCCGGCCCAAAGTTCTCAT	1115 1403	312 bp	NM_009194
NKCC1 (nested)	se 5′-TTGGAGCCATTACAGTCGTGATTC as 5′-AACCCTTGGGCTTCTTGCTCTC	1217 1354	159 bp	
S100β	se 5′-AGGCCATGGTTGCCCTCATTGAT as 5′-ACTCATGGCAGGCCGTGGTCA	17 242	246 bp	NM_009115
S100β (nested)	se 5′-TACTCCGGGCGAGAGGGTGACAA as 5′-GGCGACGAAGGCCATGAACTC	52 216	186 bp	
Synaptophysin	se 5′-AGGTGCTGCAGTGGGTCTTT as 5′-GTCTGGCGGCACATAGGCATCT	71 521	472 bp	BC014823
Synaptophysin (nested)	se 5′-CGGCTGAGCGTGGAGTGTGC as 5′-AGGGCCCCCATGGAGTAGAGGAA	136 328	215 bp	

Position 1 is the first nucleotide of the initiation codon. ‘se’ and ‘as’ mark sense and antisense primers. The sense and antisense primers were located on different exons.

### Statistical analysis

Statistical analysis was performed with Excel (Microsoft) and SPSS (IBM, Armonk, NY, USA). Normal distribution was verified using the Kolmogorov-Smirnov test before one-way ANOVA and Student’s t-test and Mauchly’s test for sphericity was used for repeated-measures ANOVA. E_GABA_ values were analyzed using an ANOVA with *post hoc* analysis by Student’s t-test. A paired Student’s t-test was used for comparisons made within the same recording, an unpaired Student’s t-test for comparisons between two different groups and repeated-measures ANOVA for comparisons of changes over time. Data are presented as mean ± SEM. Statistical significance was accepted if p < 0.05.

## Electronic supplementary material

Supplementary figures

## Data Availability

The datasets generated and analyzed during the current study are available from the corresponding author on reasonable request.

## References

[1] Pinault D (2004). The thalamic reticular nucleus: Structure, function and concept. Brain Res. Rev..

[2] Llinás RR, Ribary U, Jeanmonod D, Kronberg E, Mitra PP (1999). Thalamocortical dysrhythmia: A neurological and neuropsychiatric syndrome characterized by magnetoencephalography. Proc. Natl. Acad. Sci. USA.

[3] Herd MB, Brown AR, Lambert JJ, Belelli D (2013). Extrasynaptic GABAA Receptors Couple Presynaptic Activity to Postsynaptic Inhibition in the Somatosensory Thalamus. J. Neurosci..

[4] Steriade M (2005). Sleep, epilepsy and thalamic reticular inhibitory neurons. Trends Neurosci..

[5] Huguenard JR, McCormick DA (2007). Thalamic synchrony and dynamic regulation of global forebrain oscillations. Trends Neurosci..

[6] Blaesse P, Airaksinen MS, Rivera C, Kaila K (2009). Cation-Chloride Cotransporters and Neuronal Function. Neuron.

[7] Ben-Ari Y, Khalilov I, Kahle KT, Cherubini E (2012). The GABA excitatory/inhibitory shift in brain maturation and neurological disorders. Neuroscientist.

[8] Blaesse P, Schmidt T (2015). K-Cl cotransporter KCC2–a moonlighting protein in excitatory and inhibitory synapse development and function. Pflügers Arch. - Eur. J. Physiol..

[9] Kaila K, Price TJ, Payne JA, Puskarjov M, Voipio J (2014). Cation-chloride cotransporters in neuronal development, plasticity and disease. Nat. Rev. Neurosci..

[10] Watanabe M, Fukuda A (2015). Development and regulation of chloride homeostasis in the central nervous system. Front. Cell. Neurosci..

[11] Rivera C (1999). The K+/Cl− co-transporter KCC2 renders GABA hyperpolarizing during neuronal maturation. Nature.

[12] Yamada J (2004). Cl− uptake promoting depolarizing GABA actions in immature rat neocortical neurones is mediated by NKCC1. J. Physiol..

[13] Blaesse P (2006). Oligomerization of KCC2 Correlates with Development of Inhibitory Neurotransmission. J. Neurosci..

[14] Ulrich D, Huguenard JR (1997). Nucleus-specific chloride homeostasis in rat thalamus. J. Neurosci..

[15] Mòdol L, Cobianchi S, Navarro X (2014). Prevention of NKCC1 phosphorylation avoids downregulation of KCC2 in central sensory pathways and reduces neuropathic pain after peripheral nerve injury. Pain.

[16] Kahle KT (2014). Genetically encoded impairment of neuronal KCC2 cotransporter function in human idiopathic generalized epilepsy. EMBO Rep..

[17] Puskarjov M (2014). A variant of KCC2 from patients with febrile seizures impairs neuronal Cl− extrusion and dendritic spine formation. EMBO Rep..

[18] Saitsu H (2016). Impaired neuronal KCC2 function by biallelic SLC12A5 mutations in migrating focal seizures and severe developmental delay. Sci. Rep..

[19] Woo NS (2002). Hyperexcitability and epilepsy associated with disruption of the mouse neuronal-specific K-Cl cotransporter gene. Hippocampus.

[20] Glykys J (2009). Differences in Cortical versus Subcortical GABAergic Signaling: A Candidate Mechanism of Electroclinical Uncoupling of Neonatal Seizures. Neuron.

[21] Gagnon M (2013). Chloride extrusion enhancers as novel therapeutics for neurological diseases. Nat. Med..

[22] Silayeva L (2015). KCC2 activity is critical in limiting the onset and severity of status epilepticus. Proc. Natl. Acad. Sci. USA.

[23] Sivakumaran S (2015). Selective Inhibition of KCC2 Leads to Hyperexcitability and Epileptiform Discharges in Hippocampal Slices and *In Vivo*. J. Neurosci..

[24] Li L (2016). Chloride Homeostasis Critically Regulates Synaptic NMDA Receptor Activity in Neuropathic Pain. Cell Rep..

[25] Chen L (2017). KCC2 downregulation facilitates epileptic seizures. Sci. Rep..

[26] Ferrini F (2017). Enhancing KCC2 function counteracts morphine-induced hyperalgesia. Sci. Rep..

[27] Löscher W, Puskarjov M, Kaila K (2013). Cation-chloride cotransporters NKCC1 and KCC2 as potential targets for novel antiepileptic and antiepileptogenic treatments. Neuropharmacology.

[28] Puskarjov M, Kahle KT, Ruusuvuori E, Kaila K (2014). Pharmacotherapeutic targeting of cation-chloride cotransporters in neonatal seizures. Epilepsia.

[29] Kahle KT (2016). The KCC2 Cotransporter and Human Epilepsy: Getting Excited About Inhibition. Neurosci..

[30] Ben-Ari Y (2017). NKCC1 Chloride Importer Antagonists Attenuate Many Neurological and Psychiatric Disorders. Trends Neurosci..

[31] Moore YE, Kelley MR, Brandon NJ, Deeb TZ, Moss SJ (2017). Seizing Control of KCC2: A New Therapeutic Target for Epilepsy. Trends in Neurosciences.

[32] Szabadics J (2006). Excitatory Effect of GABAergic Axo-Axonic Cells in Cortical Microcircuits. Science (80-.)..

[33] Khirug S (2008). GABAergic Depolarization of the Axon Initial Segment in Cortical Principal Neurons Is Caused by the Na-K-2Cl Cotransporter NKCC1. J. Neurosci..

[34] Rinetti-Vargas G, Phamluong K, Ron D, Bender KJ (2017). Periadolescent Maturation of GABAergic Hyperpolarization at the Axon Initial Segment. Cell Rep..

[35] Barthó P, Payne JA, Freund TF, Acsády L (2004). Differential distribution of the KCl cotransporter KCC2 in thalamic relay and reticular nuclei. Eur. J. Neurosci..

[36] Sun Y-G (2012). GABAergic synaptic transmission triggers action potentials in thalamic reticular nucleus neurons. J. Neurosci..

[37] Shu Y, McCormick DA (2002). Inhibitory interactions between ferret thalamic reticular neurons. J. Neurophysiol..

[38] Klein PM (2018). Tenuous Inhibitory GABAergic Signaling in the Reticular Thalamus. J. Neurosci..

[39] Bazhenov M, Timofeev I, Steriade M, Sejnowski TJ (1999). Self-sustained rhythmic activity in the thalamic reticular nucleus mediated by depolarizing GABAA receptor potentials. Nat. Neurosci..

[40] Payne JA, Stevenson TJ, Donaldson LF (1996). Molecular characterization of a putative K-Cl cotransporter in rat brain. A neuronal-specific isoform. J. Biol. Chem..

[41] Hübner CA, Lorke DE, Hermans-Borgmeyer I (2001). Expression of the Na-K-2Cl-cotransporter NKCC1 during mouse development. Mech. Dev..

[42] Kanaka C (2001). The differential expression patterns of messenger RNAs encoding K-Cl cotransporters (KCC1,2) and Na-K-2Cl cotransporter (NKCC1) in the rat nervous system. Neuroscience.

[43] Matthias K (2003). Segregated expression of AMPA-type glutamate receptors and glutamate transporters defines distinct astrocyte populations in the mouse hippocampus. J. Neurosci..

[44] Höft S, Griemsmann S, Seifert G, Steinhäuser C (2014). Heterogeneity in expression of functional ionotropic glutamate and GABA receptors in astrocytes across brain regions: insights from the thalamus. Philos. Trans. R. Soc. B Biol. Sci..

[45] Khirug S (2005). Distinct properties of functional KCC2 expression in immature mouse hippocampal neurons in culture and in acute slices. Eur. J. Neurosci..

[46] Achilles K (2007). Kinetic properties of Cl uptake mediated by Na+-dependent K+-2Cl cotransport in immature rat neocortical neurons. J. Neurosci..

[47] Sipilä ST, Schuchmann S, Voipio J, Yamada J, Kaila K (2006). The cation-chloride cotransporter NKCC1 promotes sharp waves in the neonatal rat hippocampus. J. Physiol..

[48] Witte M (2014). Depolarizing chloride gradient in developing cochlear nucleus neurons: Underlying mechanism and implication for calcium signaling. Neuroscience.

[49] Taylor-Burds C, Cheng P, Wray S (2015). Chloride Accumulators NKCC1 and AE2 in Mouse GnRH Neurons: Implications for GABAA Mediated Excitation. PLoS One.

[50] Zhang L-L, Delpire E, Vardi N (2007). NKCC1 Does Not Accumulate Chloride in Developing Retinal Neurons. J Neurophysiol.

[51] Rahmati N, Hoebeek FE, Peter S, De Zeeuw CI (2018). Chloride Homeostasis in Neurons With Special Emphasis on the Olivocerebellar System: Differential Roles for Transporters and Channels. Front. Cell. Neurosci..

[52] Lein ES (2007). Genome-wide atlas of gene expression in the adult mouse brain. Nature.

[53] Deleuze C, Huguenard JR (2006). Distinct electrical and chemical connectivity maps in the thalamic reticular nucleus: potential roles in synchronization and sensation. J. Neurosci..

[54] Kahle KT (2008). Roles of the cation-chloride cotransporters in neurological disease. Nat. Clin. Pract. Neurol..

[55] Wang F (2017). NKCC1 up-regulation contributes to early post-traumatic seizures and increased post-traumatic seizure susceptibility. Brain Struct. Funct..

[56] Nolte C (2001). GFAP promoter-controlled EGFP-expressing transgenic mice: a tool to visualize astrocytes and astrogliosis in living brain tissue. Glia.

[57] Zobeiri, M. *et al*. Modulation of thalamocortical oscillations by TRIP8b, an auxiliary subunit for HCN channels. *Brain Struct. Funct*. 1–28 10.1007/s00429-017-1559-z (2017). 10.1007/s00429-017-1559-z PMC5869905 29168010

[58] Blaesse P (2015). μ-Opioid Receptor-Mediated Inhibition of Intercalated Neurons and Effect on Synaptic Transmission to the Central Amygdala. J. Neurosci..

[59] Kyrozis A, Reichling DB (1995). Perforated-patch recording with gramicidin avoids artifactual changes in intracellular chloride concentration. J. Neurosci. Methods.

