# Supplementary material for: Differential regulation of chloride homeostasis and GABAergic transmission in the thalamus
Source: Sci Rep. 2018 Sep 17;8:13929. doi: 10.1038/s41598-018-31762-2 (PMC6141474; doi:10.1038/s41598-018-31762-2)
Supplement: Supplementary file 1 — Supplementary figures [file 41598_2018_31762_MOESM1_ESM.pdf]

Differential regulation of chloride homeostasis and GABAergic transmission in the thalamus

Tobias Schmidt, Nikoo Ghaffarian, Camille Philippot, Gerald Seifert, Christian Steinhäuser, Hans-Christian Pape, Peter Blaesse

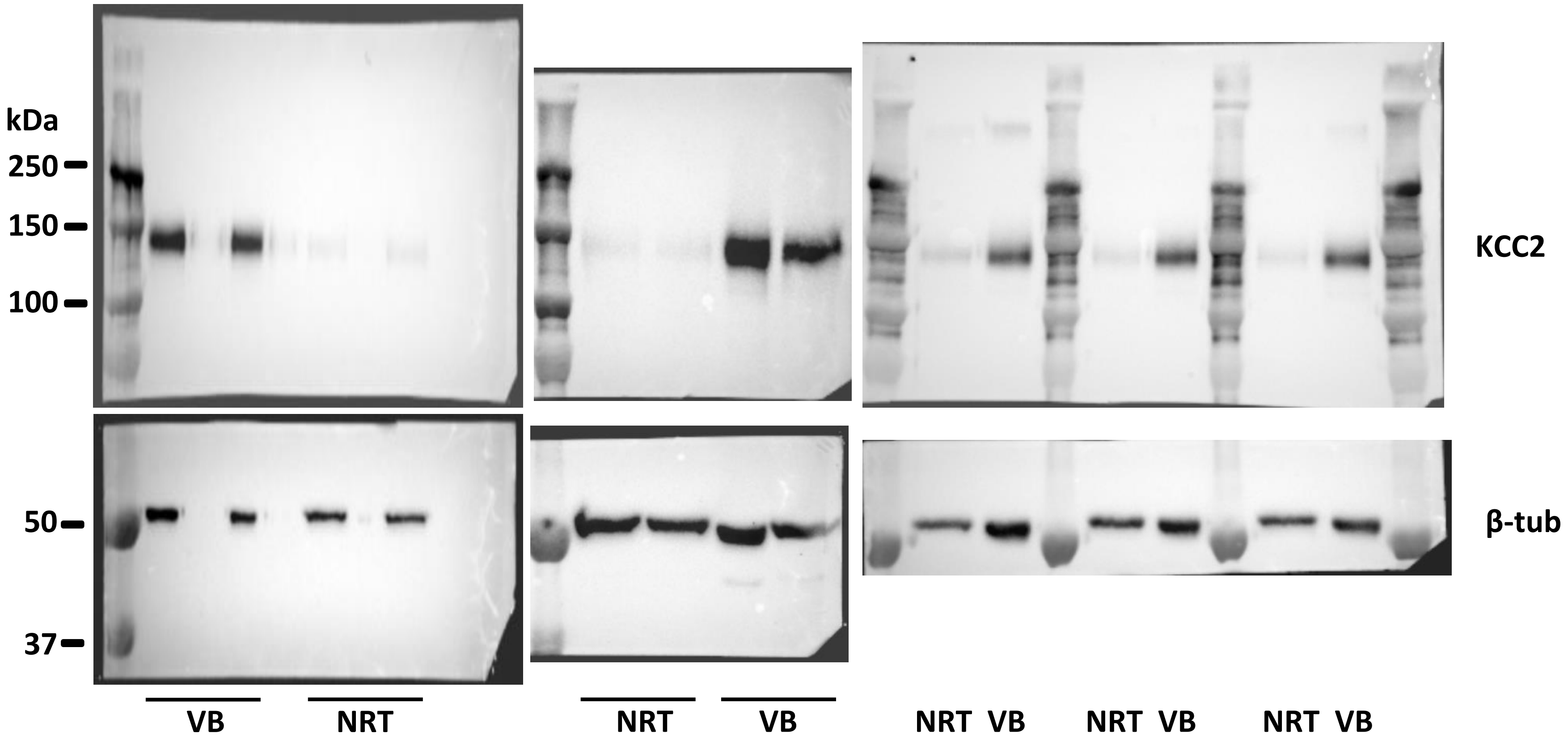

Fig. S1: Original Western Blot. Membranes were divided at ~75 kDa.
KCC2 was present in immunoblots with samples of VB tissue, but almost absent in NRT samples. β-tubulin (β-tub) was used as loading control. VB and NRT samples were collected pairwise.

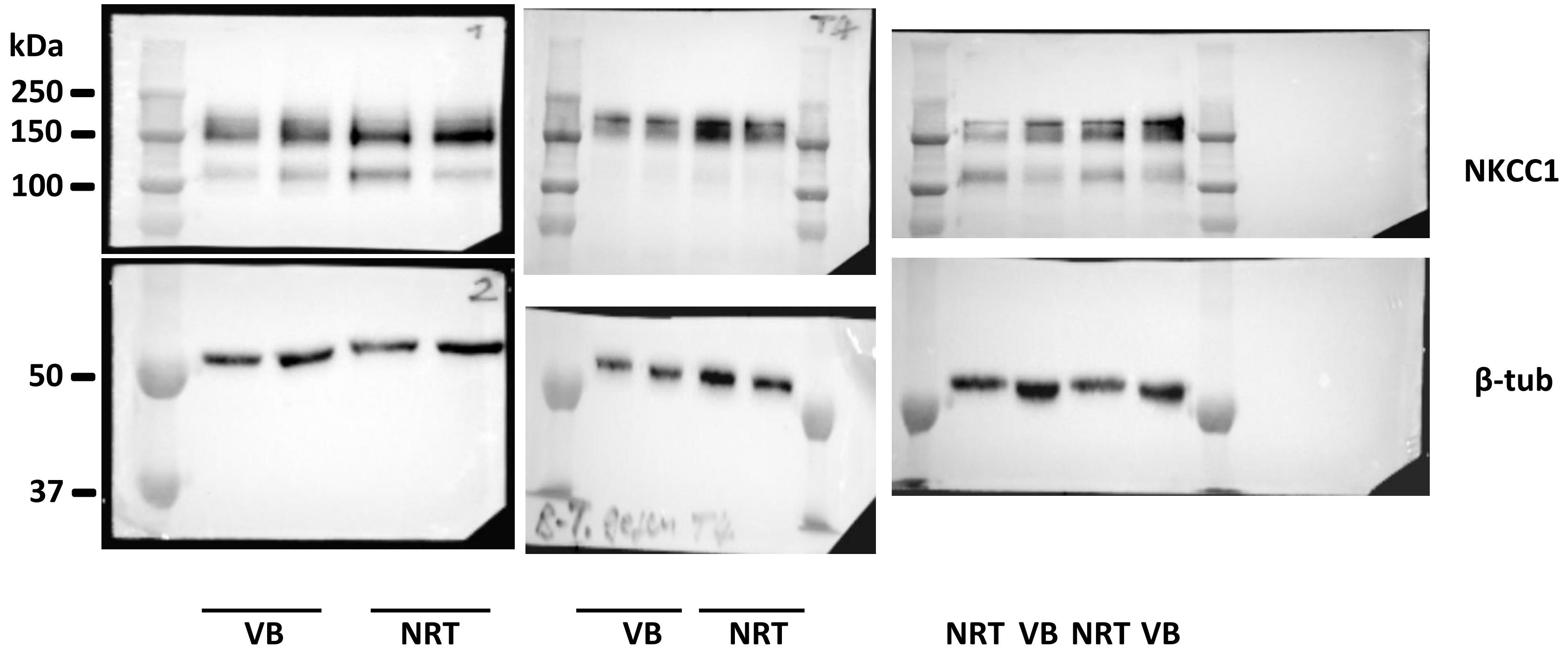

Fig. S2: Original Western Blot. Membranes were divided at ~75 kDa.
NKCC1 was expressed ubiquitously in VB and NRT. β-tubulin (β-tub) was used as loading control. VB and NRT samples were collected pairwise.

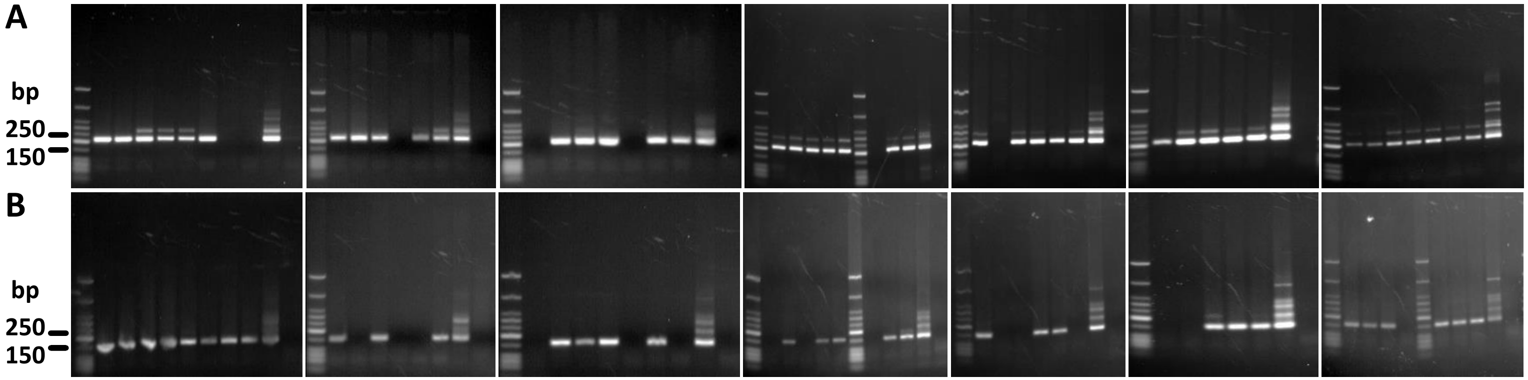

Fig. S3: Single-cell RT-PCR gels of NRT neurons **A** PCR with cytoplasm harvested from single NRT cells resulted in a PCR product (215 bp) for the neuronal marker synaptophysin in 40 samples. **B** The great majority (30) of these 40 neurons was positive for NKCC1 (159 bp PCR product). In each gel shown, a positive control (mouse brain total RNA) and a negative control (H_2O) were loaded in the second last and last lane of the gel, respectively.

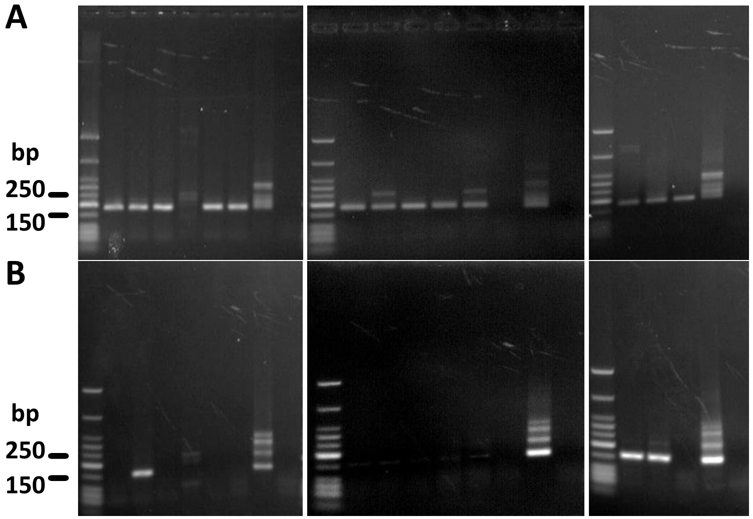

Fig. S4: Single-cell RT-PCR gels of NRT astrocytes. **A** PCR with cytoplasm harvested from single NRT cells resulted in a PCR product (186 bp) for the astrocytic marker S100β in 13 cells. **B** The NKCC1 PCR product (159 bp) was present in 3 of the samples positive for S100β. In each gel shown, a positive control (mouse brain total RNA) and a negative control (H_2O) were loaded in the second last and last lane of the gel, respectively.
